# Saliency-Aided Online RPCA for Moving Target Detection in Infrared Maritime Scenarios

**DOI:** 10.3390/s23146334

**Published:** 2023-07-12

**Authors:** Osvaldo Pulpito, Nicola Acito, Marco Diani, Gabriele Ferri, Raffaele Grasso, Dimitris Zissis

**Affiliations:** 1 Department of Information Engineering, University of Pisa, 56122 Pisa, Italy; 2 Naval Support and Experimentation Centre, Italian Naval Academy, 57127 Livorno, Italy; 3 Italian Naval Academy, Italian Navy, 57127 Livorno, Italy; 4 NATO Science and Technology Organization, Centre for Maritime Research and Experimentation, 19126 La Spezia, Italy; gabriele.ferri@cmre.nato.int (G.F.);; 5 Department of Product & Systems Design Engineering, University of the Aegean, A 1.7.1 Hermoupoli, GR84100 Syros, Greece

**Keywords:** automatic surveillance, real time, moving target detection, maritime scenario, naval targets, infrared images, data driven, machine learning, robust principal component analysis, saliency

## Abstract

Moving target detection (MTD) is a crucial task in computer vision applications. In this paper, we investigate the problem of detecting moving targets in infrared (IR) surveillance video sequences captured using a steady camera in a maritime setting. For this purpose, we employ robust principal component analysis (RPCA), which is an improvement of principal component analysis (PCA) that separates an input matrix into the following two matrices: a low-rank matrix that is representative, in our case study, of the slowly changing background, and a sparse matrix that is representative of the foreground. RPCA is usually implemented in a non-causal batch form. To pursue a real-time application, we tested an online implementation, which, unfortunately, was affected by the presence of the target in the scene during the initialization phase. Therefore, we improved the robustness by implementing a saliency-based strategy. The advantages offered by the resulting technique, which we called “saliency-aided online moving window RPCA” (S-OMW-RPCA) are the following: RPCA is implemented online; along with the temporal features exploited by RPCA, the spatial features are also taken into consideration by using a saliency filter; the results are robust against the condition of the scene during the initialization. Finally, we compare the performance of the proposed technique in terms of precision, recall, and execution time with that of an online RPCA, thus, showing the effectiveness of the saliency-based approach.

## 1. Introduction

Automatic surveillance is a vast field which is expanding more and more and deepening its capabilities and applications due to the availability of low-cost sensors on the market and to an increase in computational capabilities. Recently, there have been many studies conducted on object detection [1,2]. In this study, we focus on a maritime scenario that presents some challenges which are mostly related to the non-stationarity of the background and to the changes in lighting conditions. An infrared (IR) band is used that tends to limit the effect due to clutter motion (especially in the long wavelength band); however, extraction of the foreground, which is intended as the target, is still a difficult task. In such a non-stationary background condition, it is usually possible to refer to frame-based techniques as well as dynamic background subtraction techniques. Frame-based techniques take advantage of the spatial features within a single frame to highlight the portions of an image that are likely to attract the attention of the observer. There are many methods described in the literature that are usually based on contrast [3,4,5], on the emulation of center-surround mechanism characteristics of the human retina [5,6,7], on spatial frequency analysis [5,8], as well as on neural networks [9]. Additionally, dynamic background subtraction techniques exploit the temporal features and can be classified into frame difference methods [10,11,12,13,14,15], statistical methods such as single Gaussian [16] and Gaussian mixture model (GMM) [15,17,18], domain transform-based techniques such as fast Fourier transform [19] and wavelet transform [20,21], machine learning models such as principal component analysis (PCA) [22,23,24] and robust principal component analysis (RPCA) [25,26,27,28,29,30], optical flow [31], and neural networks and deep learning techniques [32,33,34,35,36,37]. Frame difference methods assume that the background is unimodal and provide good performance only when the scene is really steady. Statistical methods are more robust to changes in the background. Domain transform-based techniques isolate the anomalies in the sequences by taking advantage of the characteristics of the transformed domain. Machine learning models consider the multimodality of the sequences and adapt to the specific background. Optical flow follows the changes in the gradient of the signal and it is robust against camera motion, but very time consuming; therefore, it is mainly used for tracking few regions instead of detection. Neural networks and deep learning techniques are more recent techniques which are promising, but require significant quantities of labeled data for training, which, in the specific field of marine infrared video, so far, have not been easily found.

**Figure 1:**
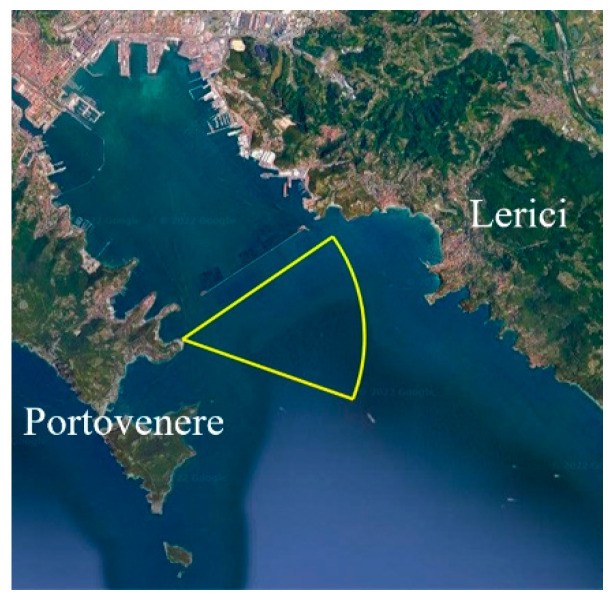
Area of operations of the experimental activity trial (WGS84 Coordinates: 44.062057° N, 9.852161° E).

In this paper, we describe, in detail, a RPCA-based technique, which is an unsupervised data-driven method, first, by introducing the simplest batch implementation, and then moving forward to a real-time approach by evaluating the online RPCA approach [38,39,40]. After considering the limits of the online implementation, we present an improved version of the method that exploits the information obtained by a saliency extraction algorithm. The idea of incorporating a saliency map into the RPCA cost function was inspired by the work of Oreifej et al. [41]. The novelty in the proposed strategy, which we call saliency-aided online moving window RPCA (S-OMW-RPCA), is the implementation of RPCA based on saliency maps in an online fashion to pursue real-time moving target detection in video sequences. We test the abovementioned technique with two saliency algorithms, namely spectral residual (SR) [8] and fine grained (FG) [7]. The choice was essentially driven by the noticeable speed of the mentioned techniques, which did not significantly increase the overall computational load. The last contribution of this paper is the evaluation of the proposed technique compared with a broadly used technique based on GMM [18], on a vast dataset of operationally significant video sequences, collected by the NATO Science and Technology Organization, Centre for Maritime Research and Experimentation (NATO STO-CMRE) during the Interactive Extreme-Scale Analytics and Forecasting (INFORE) project campaign led in Portovenere (La Spezia, Italy) during February 2022 [42]. The area of operation is shown in Figure 1. For the evaluation of the detection algorithms, some of the most representative sequences have been manually labeled through rectangular regions of interest (ROIs) surrounding the targets.

The remainder of this paper is organized as follows: In Section 2, we summarize the theoretical foundations of RPCA and its application as a background subtraction method, along with its online version; in Section 3, we present the modification proposed in this work; in Section 4, we present the dataset and discuss the qualitative and quantitative performance analysis; in Section 5, we provide concluding remarks.

## 2. Theoretical Framework and Related Works

### 2.1. Notation and Pixel Model

Throughout this paper, lowercase letters represent scalar variables, bold lowercase letters are used for vectors, capital letters represent matrices, and Greek letters are used for the coefficients.

**Figure 2:**
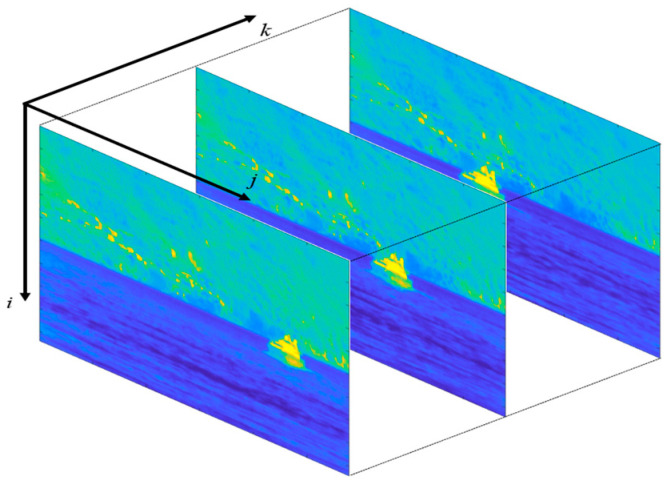
A video sequence organized in the form of a three-dimensional array. The frames are shown in false colors according to the MATLAB “parula” colormap in which the lowest values are represented in blue, and the highest values are represented in yellow.

As shown in Figure 2, the video sequences are organized in the form of three-dimensional arrays. Each element in the array represents the IR intensity value associated with the corresponding pixel. Since the IR images are monochromatic, each element carries just one value, instead of the triad of RGB videos.

Referring to [3], we model the signal $x\left(i,j,k\right)$ carried by a single pixel of spatial coordinates i and j at the quantized time instant k as:

(1) $$x\left(i,j,k\right)=l\left(i,j,k\right)+s\left(i,j,k\right)\geq 0\in R,\;i\in \left[1,h\right],\;j\in \left[1,w\right],\;k\in \left[1,n_{f}\right]$$

where $l\left(i,j,k\right)\in R$ is the background signal; $s\left(i,j,k\right)\in R$ is the target signal; h and w are the height and the width of each frame, respectively; $n_{f}$ is the number of collected frames. We also introduce the matrices $X=\left\{x_{1},\ldots ,x_{n_{f}}\right\}\in R^{n_{p}\times n_{f}}$, $L=\left\{l_{1},\ldots ,l_{n_{f}}\right\}\in R^{n_{p}\times n_{f}}$, and $S=\left\{s_{1},\ldots ,s_{n_{f}}\right\}\in R^{n_{p}\times n_{f}}$, in which $x_{i}\in R^{n_{p}}$, $l_{i}\in R^{n_{p}}$, and $s_{i}\in R^{n_{p}}$ denote the i-th frame, the corresponding background, and the target, respectively, reorganized in lexicographic order, while $n_{p}=h\times w$ is the number of pixels. Given such a model, the objective of target detection is to separate the target signal S from the background L. In the literature, such a task is commonly referred to as background subtraction.

### 2.2. RPCA for Background Subtraction

RPCA is a well-known technique that improves PCA [43] by making it robust against outliers. In fact, while PCA can be used to effectively purge the input matrix from the additive white Gaussian noise, it fails in detecting outliers. In the case of MTD, according to the previously introduced model, the input matrix X, which is representative of the input video, can be seen as the sum of a background matrix, represented by L, and an outlier matrix S, which represents the target. The idea behind using RPCA is that L is low rank, while S is sparse. Mathematically, the problem can be reformulated as that of finding L and S that satisfy Equation (2):

(2) $$\underset{L,S}{\min}\left(rank\left(L\right)+\lambda {\left\|S\right\|}_{0}\right)\;subject\;to\;X=L+S$$

where ${\left\|S\right\|}_{0}$ denotes the *l*0-pseudo-norm, which counts the total number of non-zero elements in the matrix S, while λ is a regularization parameter. Since both $rank\left(L\right)$ and ${\left\|S\right\|}_{0}$ are non-convex, the problem is not tractable as it is. For this reason, a convex relaxation makes it possible to find the optimal L and S with high probability. Such relaxation is given in Equation (3):

(3) $$\underset{L,S}{\min}\left({\left\|L\right\|}_{*}+\lambda {\left\|S\right\|}_{1}\right)\;subject\;to\;X=L+S$$

which is further relaxed in:

(4) $$\underset{L,S}{\min}\left(\frac{\mu }{2}{\left\|X-L-S\right\|}_{F}^{2}+{\left\|L\right\|}_{*}+\lambda {\left\|S\right\|}_{1}\right)\;subject\;to\;X=L+S$$

where ${\left\|L\right\|}_{*}=trace\left(\sqrt{L^{T}L}\right)$ is the nuclear norm of L, which is a convex envelope of the function $rank\left(L\right)$; ${\left\|S\right\|}_{1}=\sum_{l=1}^{H\cdot W}\sum_{k=1}^{N}\left|s\left(l,k\right)\right|$ is the *l*1-norm of S, which is a convex approximation of the *l*0-pseudo norm which promotes sparsity; as well, μ is another regularization parameter which, along with λ, controls the balance of the three terms. The convex problem in Equation (3) is known as principal component pursuit (PCP); it converges to the problem in Equation (2) and can be solved using an augmented Lagrange multiplier (ALM) algorithm [25,44]. The implementation is exposed in Algorithm 1.
**Algorithm 1:** RPCA by ALM1Input:$X=\left\{x_{1},\ldots ,x_{n_{f}}\right\}$ (observed data)μ, λ (regularization parameters)2Initialize: $S_{\left(0\right)}\leftarrow 0\in R^{n_{p}\times n_{f}}$, $Y_{\left(0\right)}\leftarrow 0\in R^{n_{p}\times n_{f}}$3**while** *not converged* **do**4(1)L(k)←D1μX−S(k−1)−1μY(k−1)5(2)S(k)←SλμX−L(k)−1μY(k−1)6(3)$Y_{\left(k\right)}\leftarrow Y_{\left(k-1\right)}+\mu \left(X-L_{\left(k\right)}-S_{\left(k\right)}\right)$7**return:**$L=L_{\left(k\right)}$ (low rank data matrix)$S=S_{\left(k\right)}$ (sparse outlier matrix)

In Algorithm 1:

- SλρY=sgnYmaxY−λρ,0 denotes the shrinkage operator applied on the matrix Y, which is the proximal operator for the *l*1-norm minimization problem $\underset{X}{argmin}\left({\frac{\rho }{2}{\left\|X-Y\right\|}_{2}^{2}+\lambda \left\|\left(X\right)\right\|}_{1}\right)$[45];
- >DλρY=USλρΣVT denotes the singular value thresholding operator applied on the matrix Y, whose singular value decomposition (SVD) is $Y=U\Sigma V^{T}$, which is the proximal operator for the nuclear-norm minimization problem $\underset{X}{argmin}\left({\frac{\rho }{2}{\left\|X-Y\right\|}_{2}^{2}+\lambda \left\|X\right\|}_{*}\right)$ [45].

RPCA is usually implemented in a batch form. In this implementation, the video is divided into batches of fixed length of $n_{win}$ frames and RPCA is applied on each batch. The length of the batches has to be chosen taking into consideration the minimum speed of the target we are interested in as well as the stationarity of the background. This method is affected by non-causality, and therefore, it does not meet the real-time requirements. In fact, we would need to wait for the collection of the entire batch before obtaining background and target estimates. A possible solution is to apply a sliding window to the input video, resulting in a moving window RPCA (MW-RPCA) [40] which, for each new collected frame, calculates the batch RPCA on the last $n_{win}$ frames to provide the background/foreground separation of the last frame. This implementation in the analysis of the video sequences usually has quite a large computational burden.

### 2.3. Online Moving Window RPCA

In the literature, there are a few proposals of online RPCA implementations [38,39,40]. For this study, we referred to online moving window RPCA (OMW-RPCA) proposed by Xiao et al. [40], which is an improvement of online robust PCA via stochastic optimization (RPCA-STOC) proposed by Feng et al. [39]. We, hereinafter, summarize the ideas behind OMW-RPCA, which, by relaxing (3), solves the following problem:

(5) $$\underset{L,S}{min}\left(\frac{1}{2}{\left\|X-L-S\right\|}_{F}^{2}+{\lambda_{1}\left\|L\right\|}_{*}+\lambda_{2}{\left\|S\right\|}_{1}\right)$$

where $\lambda_{1}$ and $\lambda_{2}$ are regularization parameters. It is worth noting that, even though by dividing the three terms in (5) by $\lambda_{1}$ we could reconduct to a form that is more similar to the one in Equation (4), which is relative to batch implementation, online implementation requires a different proportion of the regularization parameters. For this reason and in order to comply with the notation used in the reference paper, we decided to keep the notations distinguished. Therefore, hereinafter, μ and λ will refer to batch RPCA, while $\lambda_{1}$ and $\lambda_{2}$ will refer to online implementation.

According to [39], the nuclear norm of L respects the relation in Equation (6), which means that, given two matrices $U\in R^{n_{p}\times r}$ and $V\in R^{r\times n_{f}}$ such that L=UV with $rank\left(L\right)\leq r$, the nuclear norm of L is always lower than $\frac{1}{2}\left({\left\|U\right\|}_{F}^{2}+{\left\|V\right\|}_{F}^{2}\right)$.

(6) $${\left\|L\right\|}_{*}=\underset{U,V}{inf}\left\{\frac{1}{2}{\left\|U\right\|}_{F}^{2}+\frac{1}{2}{\left\|V\right\|}_{F}^{2}:L=UV\right\}$$

This means that solving the minimization problem in Equation (7) by plugging (6) into (5) also solves the minimization problem in Equation (5).

(7) $$\underset{U,V,S}{min}\left(\frac{1}{2}{\left\|X-UV-S\right\|}_{F}^{2}+\frac{\lambda_{1}}{2}\left({\left\|U\right\|}_{F}^{2}+{\left\|V\right\|}_{F}^{2}\right)+\lambda_{2}{\left\|S\right\|}_{1}\right)$$

The above-depicted nuclear norm factorization is a well-established solution for online optimization problems [39,40,46,47] and is particularly elegant since U can be seen as the basis for the low-rank subspace, in which case, V would represent the coefficients of observations with respect to the basis U. Given the input matrix X, solving Equation (7) minimizes the following so called “empirical cost function”:

(8) $$f_{k}\left(U\right)≜\frac{1}{n_{win}}\sum_{i=k-n_{win}+1}^{k}l\left(x_{i},U\right)+\frac{1}{2n_{win}}{\left\|U\right\|}_{F}^{2}$$

where $l\left(x_{i},U\right)$ is the empirical loss function for each frame, which is defined as:

(9) $$l\left(x_{i},U\right)≜\underset{v,s}{min}\left(\frac{1}{2}{\left\|x_{i}-Uv-s\right\|}_{2}^{2}+\frac{\lambda_{1}}{2}{\left\|v\right\|}_{2}^{2}+\lambda_{2}{\left\|s\right\|}_{1}\right)$$

The vectors $v_{k}$ and $s_{k}$ and the matrix $U_{k}$ are updated in two steps.

First, Equation (9) is solved in $\left(x_{k},U_{k-1}\right)$, to find $v_{k}$ and $s_{k}$; then, U is updated by minimizing the following function:

(10) $$g_{k}\left(U\right)≜\frac{1}{n_{win}}\sum_{i=k-n_{win}+1}^{k}\left(\frac{1}{2}{\left\|x_{i}-Uv_{i}\right\|}_{2}^{2}+\frac{\lambda_{1}}{2}{\left\|v_{i}\right\|}_{2}^{2}+\lambda_{2}{\left\|s_{i}\right\|}_{1}\right)+\frac{1}{2n_{win}}{\left\|U\right\|}_{F}^{2}$$

whose minimum can be found in closed form:

(11) $$U_{k}=\left[\sum_{i=k-n_{win}+1}^{k}\left(x_{i}-s_{i}\right){v_{i}}^{T}\right]{\left[\left(\sum_{i=k-n_{win}+1}^{k}v_{i}{v_{i}}^{T}\right)+\lambda_{1}I\right]}^{-1}$$

which means that U can be updated by block-coordinate descent with warm restart.

The advantage of online implementation with respect to the MW-RPCA lies in the fact that, for each new frame, only Equation (9) must be minimized with respect to two vectors, which requires remarkably less time than the minimization of Equation (4) with respect to two matrices. In addition, the update of U is in closed form and does not have to be accomplished in an iterative way, therefore, adding very small computational load.

The implementation of OMW-RPCA, unfortunately, needs an initialization which provides both the estimated rank of the matrix L and the initial basis U. Such initialization, which is called the “burn-in” phase, is accomplished by applying batch RPCA on the first $n_{b}$ frames of the sequence, where $n_{b}$ is a user-specified window size that must be higher than the expected rank of the matrix L. Although we suggest reading [40] for more details, we report in Algorithm 2 the steps of OMW-RPCA.
**Algorithm 2:** Online Moving Window RPCA1Input:
$X=\left\{x_{n_{b}+1},\ldots ,x_{n_{f}}\right\}$ (observed data revealed sequentially)μ, λ (burn-in regularization parameters)$\lambda_{1},\;\lambda_{2}$ (online regularization parameters)$X^{b}=\left(x_{1},\ldots ,x_{n_{b}}\right)$ (burn-in samples)
2Initialize:
Compute batch RPCA on burn-in samples $X^{b}$ to get *r*, $L_{n_{b}}$ and $S_{n_{b}}$Compute SVD on $L_{n_{b}}$ to get $U_{n_{b}}$ and $V_{n_{b}}$$A_{0}\leftarrow 0\in R^{r\times r}$, $B_{0}\leftarrow 0\in R^{n_{p}\times r}$ (auxiliary matrices)
3**for** k=1 to $n_{b}$ **do**4$A_{k}\leftarrow A_{k-1}+v_{k}{v_{k}}^{T},$ $B_{k}\leftarrow B_{k-1}+\left(x_{k}-s_{k}\right){v_{k}}^{T}$5**for** $k=n_{b}+1$ to $n_{f}$ **do**6(4)Reveal the sample $x_{k}$7(5)Project new sample: $\left(v_{k},s_{k}\right)\leftarrow \underset{v,s}{argmin}\left(\frac{1}{2}{\left\|x_{k}-U_{k-1}v-s\right\|}_{2}^{2}+\frac{\lambda_{1}}{2}{\left\|v\right\|}_{2}^{2}+\lambda_{2}{\left\|s\right\|}_{1}\right)$8(6)$A_{k}\leftarrow A_{k-1}+v_{k}{v_{k}}^{T}-v_{k-n_{win}}{v_{k-n_{win}}}^{T},$$B_{k}\leftarrow B_{k-1}+\left(x_{k}-s_{k}\right){v_{k}}^{T}-\left(x_{k-n_{win}}-s_{k-n_{win}}\right){v_{k-n_{win}}}^{T}$9(7)Compute $U_{k}$ with $U_{k-1}$ as warm restart$U_{k}\leftarrow \underset{U}{argmin}\frac{1}{2}Tr\left[U^{T}\left(A_{k}+\lambda_{1}I\right)U\right]-Tr\left(U^{T}B_{k}\right)$10**return:**$L=\left\{L_{n_{b}},U_{n_{b}}v_{n_{b}+1},\ldots ,U_{\left(n_{f}-1\right)}v_{n_{f}}\right\},$$S=\left\{S_{n_{b}},s_{n_{b}+1},\ldots ,s_{n_{f}}\right\}$

**Figure 3:**
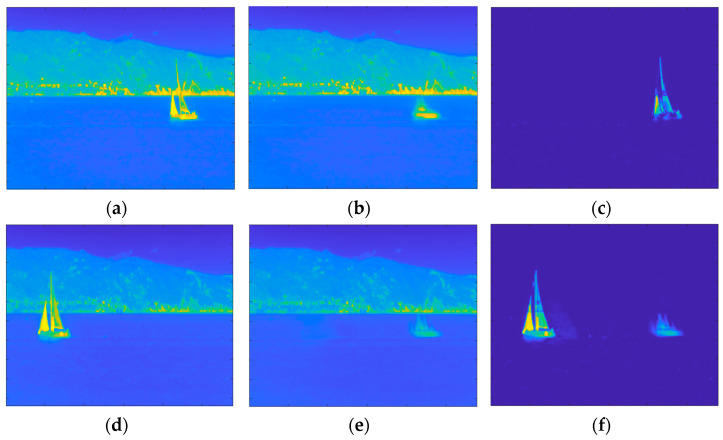
Results of OMW-RPCA applied on one scene of INFORE dataset with a burn-in of 50 frames: (**a**) Frame 25 of the sequence; (**b**) background and (**c**) foreground estimated through the burn-in phase; (**d**) Frame 349 of the sequence; (**e**) background and (**f**) foreground estimated during the online iterations. The frames are shown in false colors according to the MATLAB “parula” colormap in which the lowest values are represented in blue, and the highest values are represented in yellow.

Although OMW-RPCA solves the causality problem, the result is highly affected by the burn-in phase. In fact, in the burn-in sequence, if, on the one hand, no target is present, the successive iterations effectively isolate the target. On the other hand, if any target is present in the burn-in sequence, the successive iterations keep on considering the initial presence of the target as a part of the background. The result is that the estimated foreground and background contain a ghost of the target in the position it occupied during the burn-in phase. This problem is a sensitive issue since, in an operative context, we do not have any control of the scene during the initialization of the surveillance system. Figure 3 shows the effect of the burn-in ghosting in a sequence in which the target was present at the beginning of the recording. The upper row shows one of the first frames of the video sequence, which is included in the burn-in sequence, while the lower row shows a later frame, which is outside of the burn-in sequence. Alongside both frames, the corresponding background and foreground estimations are represented. It is worth noting that the presence of the target in the burn-in sequence affects the estimations and, even though the target is moving at a constant speed, the ghost remains in the position assumed by the boat in the burn-in sequence and does not move towards the successive positions.

A trivial idea to solve the burn-in ghosting problem is to increase the value of the regularization parameter $\lambda_{2}$, which increases the weight of ${\left\|S\right\|}_{1}$ in the loss function in Equation (5). In fact, by increasing $\lambda_{2}$, we would increase the threshold of the proximal operator associated with the *l*1-norm, which is, indeed, the shrinkage operator. By doing this, we would cut the lower intensity pixels out of the foreground. Such pixels would hopefully belong to the ghost rather than to the actual target. In this way, the background estimation would also be modified, because of the condition X=L+S, therefore, effectively deleting the ghost.

Increasing $\lambda_{2}$ is, unfortunately, an unpleasant solution for the following reasons:

- The parameter would become much more dependent on the specific input matrix X, while, in the practice, it is usually set as $\frac{1}{\sqrt{max\left(n_{p},n_{f}\right)}}$;
- Along with the ghost pixels, a higher $\lambda_{2}$ would also cause erosion of target associated pixels, affecting the detection probability as well.

In order to overcome those problems, we used a saliency-based approach, described in Section 2.4, which consisted of using a saliency map to modulate the regularization parameter associated with ${\left\|S\right\|}_{1}$.

### 2.4. Saliency-Aided RPCA

The saliency-based approach in RPCA is not new in the literature [41,48,49]. Our approach was inspired by the approach proposed by Oreifej et al. in [41], which modified the minimization problem in Equation (3) as follows:

(12) $$\underset{L,S}{min}\left({\left\|L\right\|}_{*}+\lambda {\left\|f\left(P\right)\circ S\right\|}_{1}\right)\;subject\;to\;X=L+S$$

which is then relaxed to the form:

(13) $$\underset{L,S}{min}\left(\frac{\mu }{2}{\left\|X-L-S\right\|}_{F}^{2}+{\left\|L\right\|}_{*}+\lambda {\left\|f\left(P\right)\circ S\right\|}_{1}\right)$$

where $P=\left\{p_{1},\ldots ,p_{n_{f}}\right\}\in R^{n_{p}\times n_{f}}$ is a matrix whose *i*-th column $p_{i}\in R^{n_{p}}$ is the saliency map of the *i*-th frame, scaled in the range between 0 and 1 and organized in lexicographic order. The operator ∘ indicates the element-wise multiplication, while the operator $f\left(P\right)$ denotes any function that:

- inverts the polarity of each element of P, in the sense that a low value should address high objectness confidence, and vice versa;
- scales the resulting matrix in a wider modulation range (e.g., between 0 and 20).

We use $f\left(P\right)=\beta e^{-\alpha P}$, where α and β are tuning parameters controlling the slope of the negative exponential and the dynamic of the resulting matrix, respectively. For each new frame, the saliency map is calculated through one of the many saliency filters presented in the literature. In this work, we refer to the SR and the FG algorithms because of their very small execution time. In particular, SR takes advantage of the property of the natural images known as 1/*f law*, which states that the amplitude $A\left(f\right)$ of the averaged Fourier spectrum of the ensemble of natural images obeys a distribution of the type $E\left\{A\left(f\right)\right\}\alpha 1/f$.

FG is an implementation of the well-known visual attention model, which emulates the behavior of the retina of the human eye, to highlight the spots within the image that are characterized by the highest center–surround contrast. After calculating the saliency maps, the problem in Equation (13) can be solved, again, using ALM. Referring to [41] for the details, the steps of the saliency-aided RPCA are reported in Algorithm 3.
**Algorithm 3:** Saliency aided RPCA1Input:
$X=\left\{x_{1},\ldots ,x_{n_{f}}\right\}$ (observed data)μ, λ (regularization parameters)α, β (parameters of $f\left(P\right)$)
2Initialize: $P=\left\{p_{1},\ldots ,p_{n_{f}}\right\}=0$ (empty matrix of size $\left(n_{p}\times n_{f}\right)$)3**for** k=1 to $n_{f}$ **do**4Reshape $x_{k}$ in the frame form to get the matrix $X_{k}$ of size $\left(h\times w\right)$
5Compute the saliency algorithm on the frame $X_{k}$ to get $P_{k}$
6Put $P_{k}$ in lexicographic order to get $p_{k}$ and update P
7**while** not converged **do**8(1)Lk←D1μX−Sk−1+1μYk−19(2)Sk←SλfPμX−Lk+1μYk−110(3)$Y_{\left(k\right)}\leftarrow Y_{k-1}+\mu \left(X-L_{\left(k\right)}-S_{\left(k\right)}\right)$11**return:**$L=L_{\left(k\right)}$$S=S_{\left(k\right)}$

## 3. Proposed Method: Saliency-Aided OMW-RPCA

By using a saliency-based approach, it is possible to modulate the regularization parameter controlling ${\left\|S\right\|}_{1}$ by considering the spatial features, which are not considered by RPCA. It is worth noting that, in practice, P influences the threshold of the shrinkage operator in an inverse manner. In particular, λ is increased in those zones of the frame in which the confidence of finding an object is low, while it is maintained or even decreased where saliency maps suggest the presence of foreground. Considering the solutions in Section 2.3 and Section 2.4, the problem can be formulated as:

(14) $$\underset{L,S}{\min}\left({\lambda_{1}\left\|L\right\|}_{*}+\lambda_{2}{\left\|f\left(P\right)\circ S\right\|}_{1}\right)\;subject\;to\;X=L+S$$

which is then relaxed to the form:

(15) $$\underset{L,S}{min}\left(\frac{1}{2}{\left\|X-L-S\right\|}_{F}^{2}+{\lambda_{1}\left\|L\right\|}_{*}+\lambda_{2}{\left\|f\left(P\right)\circ S\right\|}_{1}\right)$$

Plugging (6) into (15), we obtain:

(16) $$\underset{U,V,S}{min}\left(\frac{1}{2}{\left\|X-UV-S\right\|}_{F}^{2}+\frac{\lambda_{1}}{2}\left({\left\|U\right\|}_{F}^{2}+{\left\|V\right\|}_{F}^{2}\right)+\lambda_{2}{\left\|f\left(P\right)\circ S\right\|}_{1}\right)$$

Given the input matrix X, solving Equation (16) minimizes the following “saliency-enhanced empirical cost function”:

(17) $$f_{k}\left(U\right)≜\frac{1}{n_{win}}\sum_{i=k-n_{win}+1}^{k}l_{p}\left(x_{i},U\right)+\frac{1}{2n_{win}}{\left\|U\right\|}_{F}^{2}$$

where $l_{p}\left(x_{i},U\right)$ is the saliency-enhanced empirical loss function for each sample, defined as:

(18) $$l_{p}\left(x_{i},U\right)≜\underset{v,s}{min}\left(\frac{1}{2}{\left\|x_{i}-Uv-s\right\|}_{2}^{2}+\frac{\lambda_{1}}{2}{\left\|v\right\|}_{2}^{2}+\lambda_{2}{\left\|f\left(p\right)\circ s\right\|}_{1}\right)$$

The vectors $v_{k}$ and $s_{k}$ and the matrix $U_{k}$ are updated in two steps:

- First, Equation (9) is solved in $\left(x_{k},U_{k-1},p_{k}\right)$, to find $v_{k}$ and $s_{k}$;
- Then, U is updated by block-coordinate descent with warm restart.

Note that, also in this case, it is necessary to initialize the iteration with a burn-in phase. We called the described algorithm “saliency-aided online moving window RPCA” or S-OMW-RPCA. The steps of S-OMW-RPCA are detailed in Algorithm 4.
**Algorithm 4:** Saliency aided OMW-RPCA1Input:
$X=\left\{x_{n_{b}+1},\ldots ,x_{n_{f}}\right\}$ (observed data)μ, λ (burn-in regularization parameters)$\lambda_{1}$$,\;\lambda_{2}$ (online regularization parameters)α, β (parameters of $f\left(P\right)$)$X^{b}=\left(x_{1},\ldots ,x_{n_{b}}\right)$ (burn-in samples)
2Initialize:
Compute saliency aided RPCA on burn-in samples $X^{b}$ to get *r*, $L_{n_{b}}$ and $S_{n_{b}}$Compute SVD on $L_{n_{b}}$ to get $U_{n_{b}}$ and $V_{n_{b}}$$A_{0}\leftarrow 0\in R^{r\times r}$, $B_{0}\leftarrow 0\in R^{n_{p}\times r}$ (auxiliary matrices)
3**for** k=1 to $n_{b}$ **do**4$A_{k}\leftarrow A_{k-1}+v_{k}{v_{k}}^{T}$, $B_{k}\leftarrow B_{k-1}+\left(x_{k}-s_{k}\right){v_{k}}^{T}$5**for** k=1 to $n_{f}$ **do**6(1)Reveal the sample $x_{k}$7(2)Reshape $x_{k}$ in the frame form to get the matrix $X_{k}$ of size $\left(H\times W\right)$8(3)Compute the saliency algorithm on the frame $X_{k}$ to get $P_{k}$9(4)Reshape $P_{k}$ in a column vector to get $p_{k}$10(5)Project new sample: $\left(v_{k},s_{k}\right)\leftarrow \underset{v,s}{argmin}\left(\frac{1}{2}{\left\|x_{k}-U_{k-1}v-s\right\|}_{2}^{2}+\frac{\lambda_{1}}{2}{\left\|v\right\|}_{2}^{2}+\lambda_{2}{\left\|f\left(p\right)\circ s\right\|}_{1}\right)$11(6)$A_{k}\leftarrow A_{k-1}+v_{k}{v_{k}}^{\prime }-v_{k-n_{win}}{v_{k-n_{win}}}^{\prime }$,$B_{k}\leftarrow B_{k-1}+\left(x_{k}-s_{k}\right){v_{k}}^{\prime }-\left(x_{k-n_{win}}-s_{k-n_{win}}\right){v_{k-n_{win}}}^{\prime }$13(7)Compute $U_{k}$ with $U_{k-1}$ as warm restart$U_{k}\leftarrow \underset{U}{argmin}\frac{1}{2}Tr\left[U^{\prime }\left(A_{k}+\lambda_{1}I\right)U\right]-Tr\left(U^{\prime }B_{k}\right)$14**return:**$L=\left\{L_{n_{b}},U_{n_{b}}v_{n_{b}+1},\ldots ,U_{\left(n_{f}-1\right)}v_{n_{f}}\right\},$$S=\left\{S_{n_{b}},s_{n_{b}+1},\ldots ,s_{n_{f}}\right\}$

## 4. Results

### 4.1. Dataset and Qualitative Evaluation

In order to evaluate the performance of the proposed method, we used a valuable dataset containing video sequences depicting various scenarios of operational interest. In particular, the video sequences were collected by the NATO STO-CMRE during the execution of the INFORE project campaign led in the Gulf of La Spezia (Italy) during February 2022. Such a dataset includes 147 video sequences for a total of 54,363 frames. The characteristics of the sensor used to collect the sequences are shown in Table 1.

**Figure 4:**
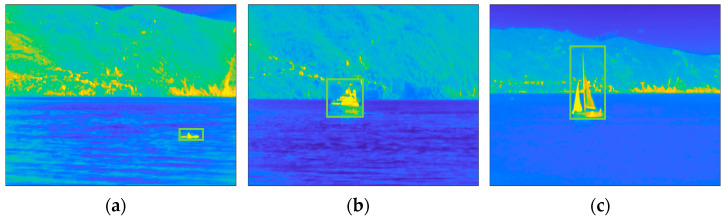

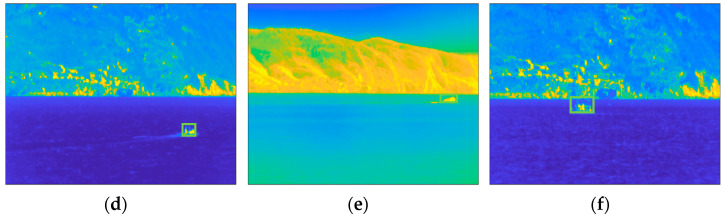
Selection of six scenes of the INFORE dataset with the relative GT ROI (green rectangles): (**a**) Kayak; (**b**) speed boat; (**c**) sailing ship; (**d**) inflatable boat; (**e**) speed boat 2; (**f**) fishing boat. The frames are shown in false colors according to the MATLAB “parula” colormap in which the lowest values are represented in blue, and the highest values are represented in yellow.

The images collected by a static camera are characterized by heterogeneous backgrounds, mainly sea, and structured. The dataset also depicts different types of targets, including boats, ships, sailing ships, kayaks, and drones, covering a wide range of size (compared with the field-of-view of the camera) and speed. For the performance evaluation, a subset of six from the most valuable sequences, for a total number of 4706 frames were manually labeled. The labels, constituting ground truth (GT), are rectangular bounding boxes surrounding the target. The algorithms aim to provide estimated ROIs in the form of rectangular bounding boxes as close as possible to the GT ROIs. Figure 4 shows one frame of each labeled sequences, along with the relative GT ROIs.

**Figure 5:**
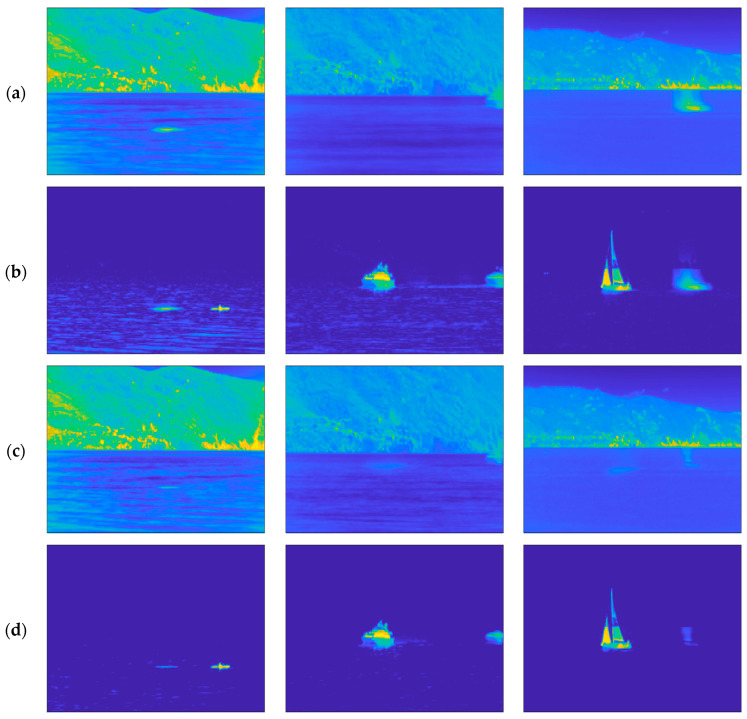

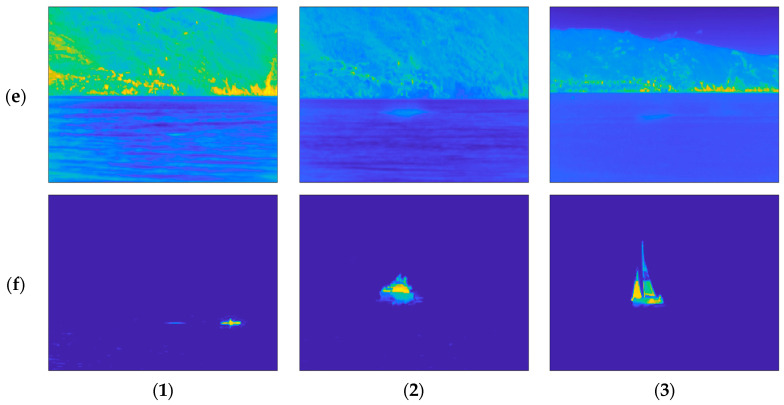
Visual comparison on background subtraction results over three scenes of the INFORE dataset. From left to right: (1) Kayak; (2) speed boat; (3) sailing boat. From top to bottom: (**a**) background and (**b**) foreground estimation via OMW-RPCA; (**c**) background and (**d**) foreground estimation via S-OMW-RPCA with FG; (**e**) background and (**f**) foreground estimation via S-OMW-RPCA with SR. The frames are shown in false colors according to the MATLAB “parula” colormap in which the lowest values are represented in blue, and the highest values are represented in yellow.

For the sake of a qualitative comparison, Figure 5 shows the background and the foreground estimation resulting from OMW-RPCA applied on the first three video sequences shown in Figure 4, as well as from the proposed techniques implemented both with SR and FG saliency filters.

Note that the size of the targets, along with their speed, as well as the warmth of the background and, in particular, the condition of the sea in all the sequences are different, which permits a more confident validation of the results. Referring to the sequences shown in Figure 5:

- The kayak is a very small target which covers 0.17% of the whole picture. The speed is such that the average permanence of the target on a single pixel is about 2 s. The background is quite hot and the waves on the sea are particularly evident.
- The speed boat is medium size and covers 0.72% of the picture. The average permanence is about 10 s. The background is colder, and the waves are less evident but still present.
- The sailing ship is an extended target which covers 2.21% of the picture. The average permanence is about 3 s. The background presents some hot spots near the horizon, while the sea is calm.

The permanence of the target on a single pixel is a very important parameter in the MTD, since targets with very long permanence may appear as still objects belonging to the background. To achieve the best performance, the MTD algorithm should be tuned on the exact speed of the object to be detected. Of course, this is not always possible since, in most cases, no a priori information about the target is available. For this reason, we want our algorithm to be effective in as many situations as possible. To match that requirement, we did not tune the algorithms on the specific video sequences. It is immediately evident that the proposed algorithm outperforms OMW-RPCA in estimating the background. In fact, the waves that affect the foreground estimation of OMW-RPCA in Figure 5b are almost completely absent in the estimation provided by the proposed technique in Figure 5d,f. Furthermore, the ghost caused by the presence of the target in the burn-in phase is greatly reduced. In particular, S-OMW-RPCA with FG has a slightly worse performance because the relative saliency map highlights the horizon regions as well, therefore, deceiving the detector in those regions.

For the sake of completeness, the values of the parameters used for the tests are listed in Table 2.

The choice of the values of $n_{b}$ was driven by the operative consideration of having the first result after a couple of seconds after turning on the system, which, considering the frame rate of the used camera, whose characteristics are listed in Table 1, corresponds to exactly 50 frames. The choice of α and β was driven by the following considerations:

- After some experiments, we noted that the ghosts of the target were effectively deleted by increasing $\lambda_{2}$ by a factor higher than 10;
- The original dynamic of the saliency map is in the range $\left[0,1\right]$ and we can assume that the lowest values (i.e., $\left[0,3.3\right]$) address the background, the middle values (i.e., $\left[3.3,6.6\right]$) indicate an uncertainty area, while the highest values (i.e., $\left[6.6,1\right]$) address the targets.

**Figure 6:**
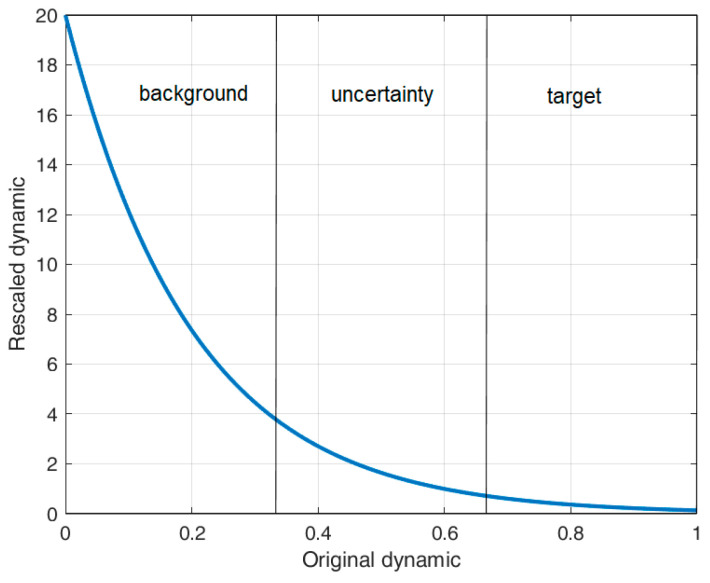
Graph of the scaling function with α=5 and β=20.

We, therefore, need to find a couple (α,β) such that the values of the rescaled dynamic which correspond to the first third are much higher than the unity, the values that correspond to the second third are roughly unitary, while the values corresponding to the last third should tend to zero. With α=5 and β=20, we obtain the scaling function graphed in Figure 6, which matches the above-reported considerations. To clarify, in order to adhere to our self-imposed requirement of not fine-tuning the algorithms, we did not engage in any empirical parameter optimization process. Instead, our approach solely relied on the aforementioned considerations.

### 4.2. Precision-Recall Curves

For the quantitative evaluation, we refer to the precision and recall scores, which are defined in Equation (19).

(19) $$Precision=\frac{TP}{TP+FP}\;Recall=\frac{TP}{TP+FN}$$

where TP indicates the number of true positives (i.e., detected targets), FP indicates the number of false positives (i.e., false alarms), and FN indicates the number of false negatives (i.e., undetected targets).

**Figure 7:**
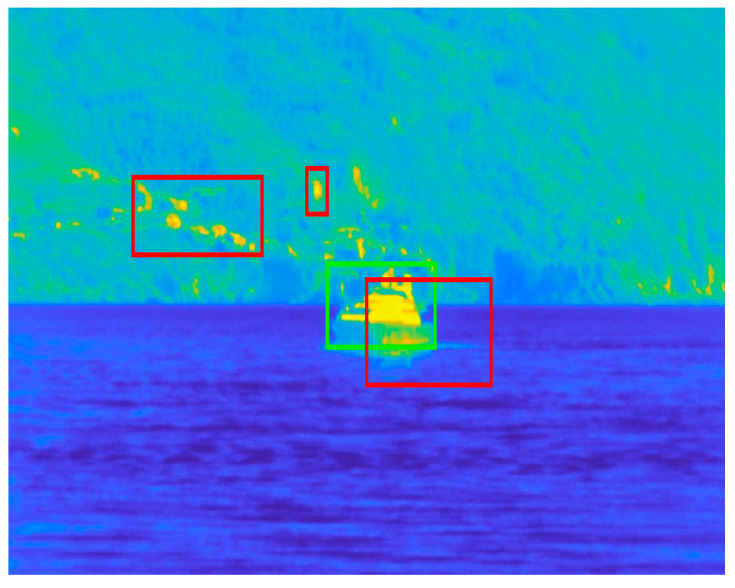
Example of ground truth ROI (green) vs. estimated ROI (red). The frames are shown in false colors according to the MATLAB “parula” colormap in which the lowest values are represented in blue, and the highest values are represented in yellow.

In order to define the TP, FP, and FN scores, let us consider Figure 7. The TP is easily represented by the area of the intersection between the GT ROI (green rectangle) and the estimated ROIs (red rectangles). The FP is represented by the area of the estimated ROIs minus the TP. Therefore, the precision denominator corresponds to the whole area of the estimated ROIs. The FN is represented by the area of the GT ROI minus the TP. Therefore, the recall denominator corresponds to the whole area of the GT ROI.

**Figure 8:**
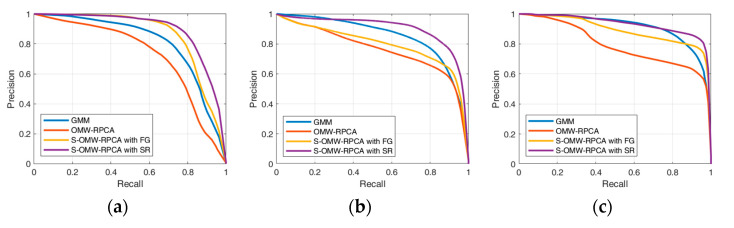

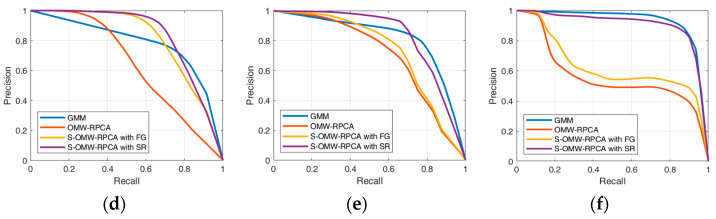
Precision/recall curves over six scenes of INFORE dataset: (**a**) Kayak; (**b**) speed boat; (**c**) sailing ship; (**d**) inflatable boat; (**e**) speed boat 2; (**f**) fishing boat.

**Figure 9:**
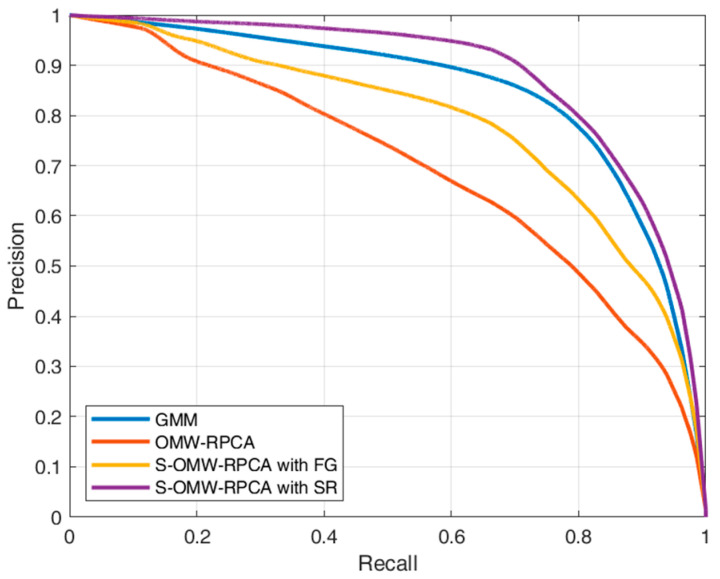
Precision/recall average curves calculated over six sequences of INFORE dataset.

Precision and recall are broadly used performance indices, which are mostly provided as a pair of values in correspondence of a preventively chosen threshold [48,49]. Such a practice is adequate when the choice of the threshold is included in the evaluation, which is not the case. For this reason, we prefer to present the results in the form of curves obtained by varying the thresholds in a range covering all the conditions from 0% to 100% of the pixels denoted as positives. This approach (which is inspired by the receiver operating characteristics, broadly used in statistical decision theory), has already been utilized in [15] and has the advantage of decorrelating the results from the choice of the threshold, which can be, therefore, demanded to any suitable decision criteria. In addition to the comparison between the proposed technique with OMW-RPCA, we also provide the results of the moving object detector based on the improved adaptive Gaussian mixture model (GMM) [18] as a benchmark. The results for the six labeled sequences are reported in Figure 8, while Figure 9 depicts the average curves.

The aim of the algorithms in terms of precision and recall is to reach the highest possible precision, given an acceptable level of recall (e.g., 0.8), which means that the curve must tend to the top-right corner of the graph. From the curves in Figure 8, it emerges that the proposed S-OMW-RPCA always outperforms the simple OMW-RPCA and, in particular, S-OMW-RPCA with SR always outperforms S-OMW-RPCA with FG. Furthermore, as compared with the MTD based on GMM, S-OMW-RPCA with SR also performs remarkably well in the first three sequences, in the fourth and fifth sequences it loses precision in correspondence of high recalls, while in the sixth sequence it provides quite similar results. Figure 9 shows the average curves, and the results confirm that S-OMW-RPCA outperforms OMW-RPCA and, on average, performs slightly better than GMM.

Finally, for a more synthetic comparison, in Table 3, we show the precision obtained by each algorithm in each sequence by setting the recall to a value of 0.8, which, when working with ROIs, is usually considered to be an acceptable result. The bold scores represent the best precision performance for each sequence on average, while the red scores represent the best performance among the RPCA-based techniques.

The results in Table 3, confirm that, for an acceptable value of recall, S-OMW-RPCA, especially the version with SR, yields much higher precision than the simple OMW-RPCA. Furthermore, compared to the widely used GMM approach, the performance of the proposed algorithm depends on the specific sequence; nonetheless, on average, it manifests the highest precision.

### 4.3. Execution Time

Regarding the execution time, as previously mentioned, the SR and FG saliency algorithms were chosen not only for their quality but also for their speed. In fact, for a 22k pixel frame, such as those of the used sequences, the average execution time of the SR and FG is 4.86 × 10^−7^ and 1.65 × 10^−5^ s per frame, respectively. Such values are several orders of magnitude lower than the execution time of OMW-RPCA, which, in the online phase is in the order of 10^−2^ s per frame. This fact may mislead one to think that the execution time of OMW-RPCA and the execution time of the proposed S-OMW-RPCA should be approximately equal. On the contrary, the different weights given by the saliency maps lead to a significant variation in the time required for the convergence of the ALM used to solve the burn-in phase, and for the minimization of Equation (18) for the solution of the online phase. The experiments were conducted using the MATLAB environment with a computer whose specifications are indicated in Table 4. The results are shown in Table 5. The bold scores represent the minimum execution time achieved by the competing algorithms for each sequence and in each phase. We would like to clarify that the algorithms used in this study were not optimized. Therefore, it is important to interpret the results solely for the purpose of comparison.

Counterintuitively, the results show that, while the speed of the ALM used to solve the burn-in phase is always increased by the use of saliency maps, the minimization of Equation (18) for the solution of the online phase is slowed down. It is worth noting that, even though the execution time of the online phase is slightly higher than the frame collection period, which is the inverse of the frame rate, an optimization of the algorithms in a leaner programming environment would easily permit real-time implementation. We are currently working on methods to distribute the computational tasks so that the execution time is minimized.

## 5. Conclusions

In this paper, we investigated the problem of online moving target detection in marine scenarios with infrared sequences captured using steady cameras. We particularly focused on RPCA, which is a data-driven technique with many fields of application, broadly used, especially in its batch implementation, for object detection. We started by analyzing the online implementation and, after considering their limitations, we proposed an improvement based on the use of a saliency map to modulate the regularization parameters of RPCA. For the saliency maps, we used the FG and SR algorithms, which are very fast saliency filters that did not significantly increase the computational load of the whole technique. Finally, we compared the performance of the proposed technique with that of the online implementation, as well as with another broadly used technique based on GMM, which serves as a benchmark for the state-of-the-art MTD techniques. The algorithms were tested through a valuable dataset of video sequences collected by NATO STO-CMRE during the INFORE Project, during February 2022, in the Gulf of La Spezia. The comparisons were conducted both qualitatively and quantitatively, the latter through the widely used metrics of precision and recall. The results showed that the proposed saliency-aided technique greatly improved online RPCA especially when the SR filter was used for saliency map extraction. Furthermore, the proposed technique also performed better than GMM, on average. The execution time was also evaluated. Specifically, S-OMW-RPCA was faster than OMW-RPCA in the burn-in phase, while it was slower in the online phase. Finally, we specified that the algorithms were not optimized with respect to their computational load, but it is our belief that a careful optimization on a leaner programming language would make the proposed algorithm suitable for real-time surveillance purposes.

## Figures and Tables

**Table 1:** Hardware specifications of the IR camera.

Detector Type	MWIR Indium Antimonide (InSb) or Mercury Cadmium Telluride (MCT)
Spectral range	3 to 5 μm
FOV	1.1(H)° × 0.84°(V) to 14.1°(H) × 10.5°(V)
IFOV	0.383 mrad to 0.031 mrad
Array format	640 × 512 pixel
Frame rate	25 fps
Thermal sensitivity	25 mK

**Table 2:** Values of the parameters used for the test.

$\mu =\frac{1}{4*mean\left(\left|X\right|\right)}$ (Default [40])
$\lambda =\frac{1}{\sqrt{max\left(n_{p},n_{f}\right)}}$ (Default [40])
$\lambda_{1}=\frac{1}{\sqrt{max\left(n_{p},n_{f}\right)}}$ (Default [40])
$\lambda_{2}=\frac{1}{\sqrt{max\left(n_{p},n_{f}\right)}}$ (Default [40])
$n_{b}=50$
α=5
β=20

**Table 3:** Precision score, measured at a recall of 0.8 for each tested algorithm (rows), over six sequences of INFORE dataset (columns), singularly and on average. The best score for each column is highlighted by using bold numbers, while the best score over the RPCA techniques is shown in red.

	Kayak	Speed Boat	Sailing Ship	Inflatable Boat	Speed Boat 2	Fishing Boat	Average
GMM	0.67	0.77	0.87	**0.65**	**0.74**	**0.93**	0.78
OMW-RPCA	0.47	0.66	0.67	0.24	0.36	0.47	0.49
S-OMW-RPCA with FG	0.77	0.71	0.82	0.52	0.40	0.53	0.63
S-OMW-RPCA with SR	**0.85**	**0.86**	**0.89**	0.59	0.64	0.91	**0.80**

**Table 4:** Hardware specifications of the computer.

Processor	Intel CPU Core I9-11900
RAM	4 × 16 GB 3600 MHz
GPU	NO

**Table 5:** Comparison between the execution time of OMW-RPCA and the proposed S-OMW-RPCA with SR and FG, both during the burn-in and the online phases. The values are expressed in seconds per frame. The best scores are highlighted by bold numbers.

		Kayak	Speed Boat	Sailing Ship	Inflatable Boat	Speed Boat 2	Fishing Boat
Burn-in phase	OMW-RPCA	0.0249	0.0208	0.0558	0.0235	0.0342	0.0258
	S-OMW-RPCA with SR	**0.0072**	**0.0032**	**0.0240**	0.0117	**0.0196**	**0.0099**
	S-OMW-RPCA with FG	0.0074	0.0033	0.0277	**0.0112**	0.0232	0.0101
Online phase	OMW-RPCA	**0.0901**	**0.071**	**0.0754**	**0.0699**	**0.0573**	**0.0749**
	S-OMW-RPCA with SR	0.1942	0.1944	0.1954	0.1977	0.192	0.1963
	S-OMW-RPCA with FG	0.1954	0.1939	0.1959	0.1952	0.1946	0.1978

## Data Availability

The data set used in this work is owned by NATO and legal restrictions apply to its redistribution. The data set can be released to NATO Nations in accordance to the NATO data exploitation framework policy. A public sample of the data set is available as “INFORE22 sea trial open data set” at the URL https://zenodo.org/record/6372728 (accessed on 6 July 2023). under terms and conditions specified in the “Conditions for use and distributions” paragraph.
